# Acceleration of Bone Regeneration in Critical-Size Defect Using BMP-9-Loaded nHA/ColI/MWCNTs Scaffolds Seeded with Bone Marrow Mesenchymal Stem Cells

**DOI:** 10.1155/2019/7343957

**Published:** 2019-04-11

**Authors:** Ran Zhang, Xuewen Li, Yao Liu, Xiaobo Gao, Tong Zhu, Li Lu

**Affiliations:** ^1^Department of Oral and Maxillofacial Surgery, School of Stomatology, China Medical University, 117 Nanjing North Street, Shenyang 110002, China; ^2^Department of Oral Anatomy and Physiology, School of Stomatology, China Medical University, 117 Nanjing North Street, Shenyang 110002, China; ^3^Department of Pediatric Dentistry, School of Stomatology, China Medical University, 117 Nanjing North Street, Shenyang 110002, China; ^4^Department of Oral and Maxillofacial Surgery, The Affiliated Hospital of Chifeng University, Chifeng 024005, China

## Abstract

Biocompatible scaffolding materials play an important role in bone tissue engineering. This study sought to develop and characterize a nano-hydroxyapatite (nHA)/collagen I (ColI)/multi-walled carbon nanotube (MWCNT) composite scaffold loaded with recombinant bone morphogenetic protein-9 (BMP-9) for bone tissue engineering by* in vitro* and* in vivo* experiments. The composite nHA/ColI/MWCNT scaffolds were fabricated at various concentrations of MWCNTs (0.5, 1, and 1.5% wt) by blending and freeze drying. The porosity, swelling rate, water absorption rate, mechanical properties, and biocompatibility of scaffolds were measured. After loading with BMP-9, bone marrow mesenchymal stem cells (BMMSCs) were seeded to evaluate their characteristics* in vitro* and in a critical sized defect in Sprague-Dawley rats* in vivo*. It was shown that the 1% MWCNT group was the most suitable for bone tissue engineering. Our results demonstrated that scaffolds loaded with BMP-9 promoted differentiation of BMMSCs into osteoblasts* in vitro* and induced more bone formation* in vivo*. To conclude, nHA/ColI/MWCNT scaffolds loaded with BMP-9 possess high biocompatibility and osteogenesis and are a good candidate for use in bone tissue engineering.

## 1. Introduction

Craniofacial bone defects are a common disease and difficult to study experimentally and treat clinically. Conventional allografts and autografts have limitations such as immune rejection, disease transmission, malunion, and flap necrosis [1, 2]. Researchers are developing new alternatives to traditional methods for bone defect regeneration [3]. The development of artificial bone transplantation has been greatly facilitated by tissue engineering. The emergence and development of tissue engineering offer tremendous potential. The properties of bio-scaffolds play an important role in bone tissue engineering [4, 5]. An ideal biomaterial for bone tissue engineering should provide biocompatibility, good surface activity, appropriate pore sizes and porosity, high mechanical strength, and plasticity [6–8].

Hydroxyapatite (HA) is considered as the most studied biomaterials because of its proven biocompatibility and being the main inorganic constituent of bone [9]. Nano-hydroxyapatite (nHA) as a biomaterial is similar to the inorganic component of human bone. Therefore, it is considered to be more suitable for bone scaffolds. It is also an important source of calcium and phosphate and very important for bone regeneration and bone remodeling [10]. Collagen I (ColI) is a major organic component of bone, which has been used for cell culture, growth, and differentiation. It is an ideal base material to fabricate a composite porous scaffold [11]. Carbon nanotubes (CNTs) are a new nano-material developed by Iijima et al. [12], known as “super fibers”, which are divided into single-walled carbon nanotubes (SWCNTs) and multi-walled carbon nanotubes (MWCNTs). In recent years, MWCNTs, which possess exceptional chemical, electrical, and mechanical properties, have been advocated for application in bone tissue engineering as reinforcement materials. They have been applied to bone tissue engineering by Zanello et al. [13] to increase the physical and chemical properties of scaffolds. In addition, Narita et al. [14] reported that MWCNTs can help to prevent the development of osteolysis around the implants. Bone morphogenetic protein-9 (BMP-9) (also known as GDF-2) has direct effects on angiogenesis, chondrogenesis, and osteogenesis [15]. Recent studies have demonstrated that BMP-9 shows the most effective osteogenic behavior among BMPs* in vivo* and* in vitro* [16–18].

Our objective was to fabricate a nHA/ColI/MWCNT (nHACM) composite scaffold for bone tissue engineering to enhance osteogenesis of bone marrow mesenchymal stem cells (BMMSCs). MWCNTs have been used to improve the mechanical properties of classical nHA/ColI scaffolds. Furthermore, a series of* in vitro* experiments were performed to characterize and determine the best concentration of MWCNTs. Then, nHACM was used as a carrier to load BMP-9. This new BMP-9-releasing nHACM composite scaffold (nHACM/B9) was fabricated and evaluated* in vitro* and* in vivo*.

## 2. Materials and Methods

### 2.1. Materials

nHA was purchased from Emperor Nano Material (Nanjing, China). ColI was purchased from Corning (NY, USA). BMMSCs from Sprague-Dawley (SD) rats were purchased from PuheBio (Wuxi, China). MWCNTs (-COOH) were purchased from Boyu Gaoke (Beijing, China). *α*-Minimum essential medium (*α*MEM), phosphate-buffered saline (PBS), trypsin, and fetal bovine serum (FBS) were purchased from HyClone (UT, USA). BMP-9 was purchased from PeproTech (NJ, USA).

### 2.2. Fabrication of Scaffolds

After freeze drying, ColI was dissolved in 50 mmol/L acetic acid, and the final concentration of ColI was adjusted to 2% (w/v). nHA was added to ColI at 3:7 (w/w) [19, 20]. After preparing various concentrations of MWCNTs (0.5, 1, and 1.5% wt), they were mixed with the nHA and ColI-blended solution [21, 22]. The pH values of the mixtures were neutralized using 0.05 M sodium hydroxide solution. nHA-ColI-MWCNT solutions were stirred at low temperature for 12 h.

The solutions were poured into Teflon culture plates and lyophilized (Alpha 1-2 LD plus, Christ, Germany) for 72 h to obtain nHACM scaffolds. After a series of scaffold evaluations, BMP-9 (500ng/ml) was loaded into the scaffolds when the solution was stirred before freeze drying [23, 24] (Supplementary Figure A). We found that the most appropriate proportion of MWCNTs was 1% for bone tissue engineering in a preliminary experiment (Supplementary Figure B). Therefore, this concentration was used in subsequent experiments* in vitro *and* in vivo*.

### 2.3. Scaffold Characterization Studies

nHACM scaffold samples were fractured, coated with gold, and observed using a scanning electron microscope (S4800, Hitachi, Japan). Pore sizes were measured in SEM images by ImageJ software. To measure pore size, scale bars were set within the SEM image, representing a known distance. The contour of the pores was outlined and measured (*μ*m). Different cross sections of the composites were assessed [25]. The porosity of scaffolds was evaluated using ethyl alcohol (EtOH) displacement. The primary volume of EtOH was V_1_. The total volume of EtOH after scaffold immersion was V_2_. The residual EtOH volume after scaffold removal was V_3_. The following formula was used to calculate porosity: Porosity (%) = (V_1_−V_3_)/(V_2_ −V_3_)×100%. Water absorption mirrored quality changes after scaffolds were immersed in PBS. W_1_ is the swollen weight and W_2_ is the dried weight. The following formula was used: water absorption rate (%) = (W_1_−W_2_)/W_2_ × 100%. The swelling property was indicated by volume changes after immersion in PBS. Wet and dried volumes were, respectively, recorded as V_a_ and V_b_. V_a_ was determined according to Archimedes' principle, and V_b_ of the scaffold was measured using a Vernier caliper. The swelling index of the scaffold was calculated using the following formula: Swelling ratio (%) = (V_a_−V_b_)/V_b_×100%. The mechanical compression of the standardized scaffolds (10 mm in diameter and 10 mm in height) was measured by a universal material testing machine (E1000, Instron, USA) with a 100 N load at room temperature. Crosshead speed was 1 mm/min.

### 2.4. Culture of BMMSCs

BMMSCs were cultured in *α*MEM with 10% FBS and 1% penicillin/streptomycin (GE Healthcare Life Sciences Hyclone Laboratory, UT, USA). The cells were cultured in 75-cm^2^ flasks at 37°C in a humidified atmosphere of 5% CO_2_ (Supplementary Figure C). The scaffolds used for cell seeding were cut with 8.00 mm in diameter × 2.00 mm height. 50 *μ*l of BMMSCs at 4 × 10^4^ cells/well were cultured on pre-wetted nHACM scaffolds for 72 h at 37°C and 5% CO_2_.

### 2.5. BMMSC Morphology on nHACM/B9 Scaffolds

After culture on scaffolds for 72 h, the morphologies of BMMSCs were observed. The samples were fixed in 2.5% glutaraldehyde (Solarbio, Beijing, China) at 4°C overnight. After dehydration and drying, SEM was used to observe the samples.

### 2.6. Counting Kit-8 (CCK-8) Assay

A CCK-8 (Beyotime, Shanghai, China) assay was used to examine the viability and activity of grafted cells after BMMSCs were cultured on nHACM and nHACM/B9 scaffolds for 1, 3, 5, and 7 days. At specific time points, the medium was removed and replaced with serum-free culture medium containing 0.5 mg/mL CCK-8 solution. Absorbance was measured at 450 nm using an enzyme-linked immunosorbent assay reader (Infinite M200, Tecan, Austria).

### 2.7. BMMSC Differentiation and Alkaline Phosphatase (ALP) Activity Assay

BMMSCs were cultured on nHACM loaded with BMP-9 and treated with 50 *μ*g/ml ascorbic acid, 5 mM *β*-glycerophosphate, and 10 nM dexamethasone to induce osteoblast differentiation. The culture medium was changed every 2 days. After induction for 1, 4, 7, and 10 days, ALP activity was determined in cell lysates by an ALP assay kit (Beyotime, Shanghai, China), and absorbance was read at 520 nm using an ELISA plate reader.

### 2.8. Quantitative Reverse Transcription-Polymerase Chain Reaction (qPCR)

Isolation and reverse transcription of total RNA were carried out. The synthesized cDNA was then used to perform real-time-PCR. Briefly; after 7 days of culture, the total RNA was extracted by using TRIzol reagent (Invitrogen, Carlsbad, CA, USA). RNA was reverse-transcribed into cDNA, which was used for real-time PCR on a LightCycler Nano (Roche, Switzerland), according to the manufacturer's instructions. The expression of osteogenic markers osteocalcin (OCN), collagen type I, alpha 1 (ColI*α*1), and osteopontin (OPN) was analyzed by PCR amplifications using specific primers (Table 1). All reactions were run in triplicate. Target genes were normalized against GAPDH as the housekeeping gene.

### 2.9. In Vivo Experiments

The* in vivo* experiments were approved by China Medical University's Animal Care and Use Committee. We used 15 male 8-week-old SD rats as experimental animals. After inducing anesthesia with chloral hydrate, skin was prepared and sterilized, and an incision on the center of the scalp was made. After exposing the cranial bone, an 8 mm critical size defect (CSD) was created with an 8 mm trephine bur. Next, scaffolds with or without cells were implanted into the defects. At the end of the operation, the incision was sutured. Rats were given 5 mg/kg carprofen post-surgery for analgesia, placed in a recovery cage, and observed until awake and ambulatory.

All rats were divided into three groups: group 1, blank control; group 2, nHACM+BMMSCs; group 3, nHACM/B9+BMMSCs. Twelve weeks after the operations, animals were euthanized by cervical dislocation under deep anesthesia with isoflurane.

### 2.10. Evaluation of Bone Regeneration

Three-dimensional images of the calvarias were captured with a multi-slice spiral computed tomography (CT) scanner (SOMATOM Definition AS+; Siemens Healthineers, Germany) at 12 weeks after implantation. After standard radiological image processing, the scanning images and CT values of calvarial bones were obtained using SyngoMMWP software. NIH ImageJ software was used to measure the area of new bone regeneration. The CT value (Hounsfield unit; HU) was determined to evaluate the density of regenerated tissue. The fixed calvarial bone samples were embedded in paraffin wax and then cut into 5-*μ*m thick sections. The sections were stained with hematoxylin and eosin (H&E), and then images were captured under a microscope (CKX41; Olympus Co., Tokyo, Japan). New bone volumes of regenerated tissue were analyzed using ImageJ software.

### 2.11. Statistical Analysis

All quantitative data are expressed as the mean ± SD of three replicates for each test. Statistical significance was analyzed by SPSS 17.0 software (SPSS Inc., Chicago, IL). Pairwise comparisons were assessed using Student's* t*-test. For all experiments, a *P* value of less than 0.05 was considered significant.

## 3. Results

### 3.1. Synthesis and Characterization of nHACM Scaffolds

**Figure 1:**
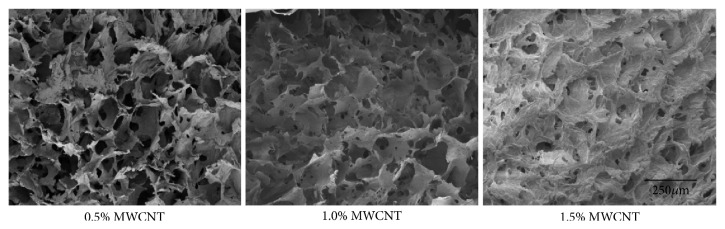
Micrographs of the scaffolds with varying MWCNT content (0.5%, 1.0%, and 1.5% wt). SEM showed that nHACM scaffolds had a porous structure with well-oriented pores enclosed by a thin wall from the surface to the inside. As the concentration of MWCNT increased, the pore size and number decreased.

**Figure 2:**
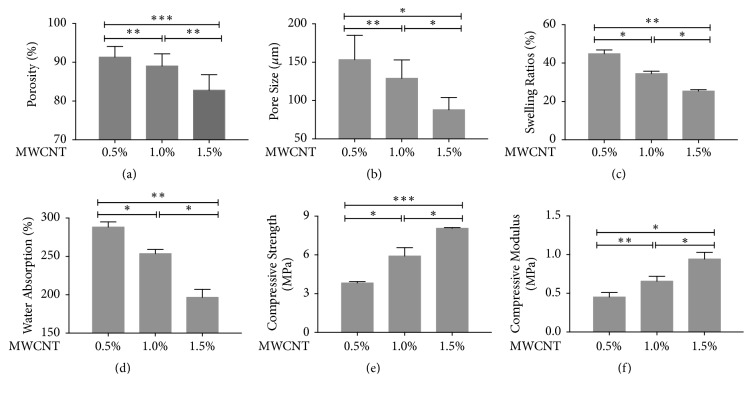
Characterization and physicochemical properties of scaffolds. (a-d) As the concentration of MWCNT increased, the pore size, porosity, water absorption, and swelling ability decreased. (e, f) Compressive strength and compressive modulus decreased with increasing MWCNT concentrations. Mean±SD; n=3; *∗p*<0.05, *∗∗p*<0.01, and *∗∗∗p*<0.001.

Ethyl alcohol displacement was used to analyze the porosity of scaffolds. The results showed that the porosity of 0.5% wt MWCNT group was 91.34±3.02%, the porosity of 1.0% wt MWCNT was 89.04±3.26%, and the porosity of 1.5% wt MWCNT was 82.82±2.74%. Scanning electron microscope was used to observe and analyze the inside of scaffolds. The results showed that nHACM scaffolds had a porous structure with well-oriented pores enclosed by a thin wall from the surface to inside (Figure 1). Most pores of the scaffolds were uniform. This structure benefits cells in going inside the scaffolds and forming attachment. An optimum scaffold should possess a suitable 3D structure and pore size for nutrient transport and preventing cell loss. By using ImageJ to analyze the image of SEM, we obtained the pore size of 0.5% wt MWCNT group was 153.40±31.17 *μ*m, the pore size of 1.0% wt MWCNT group was 129.00±23.38 *μ*m, and the pore size of 1.5% wt MWCNT group was 88.00±11.73 *μ*m. As the concentration of MWCNT increased, the porosity and pore size of scaffolds decreased (Figures 2(a) and 2(b)). Water absorption and a swelling ability are important factors in evaluating the usefulness of a composite material for bone tissue engineering. The water absorption ability of nHACM scaffolds was measured in terms of the degree of swelling at equilibrium. The degree of swelling of the scaffolds ranged from 25.4 to 44.9%, while the degree of water absorption was between 196 and 288%. As the concentration of MWCNT increased, the swelling ability and water absorption of scaffolds decreased (Figures 2(c) and 2(d)). Mechanical strength is important to the bone tissue engineering scaffolds. The compressive modulus and compressive strength decreased with the increasing concentration of MWCNT (Figures 2(e) and 2(f)). Scaffolds containing 1.5% wt MWCNT had the highest compressive modulus (8.06±0.07 MPa) and compressive strength (0.94±0.08 MPa) among the three kinds of scaffolds, but they had lowest porosity, pore size, swelling ratios, and water absorption. And scaffolds containing 0.5% wt MWCNT were opposite. So we chose 1.0% wt MWCNT in subsequent experiments.

**Figure 3:**
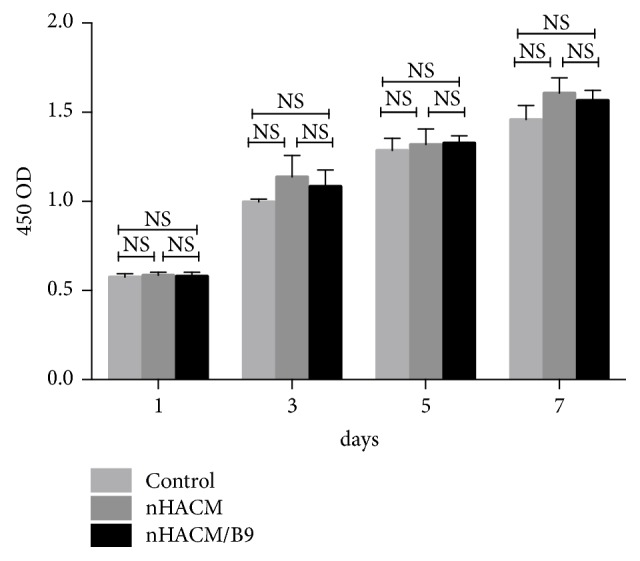
CCK-8 assays to measure numbers of BMMSCs on nHACM and nHACM/B9 scaffolds. No statistically significant differences were seen among the three groups. Mean±SD; n=3.

**Figure 4:**
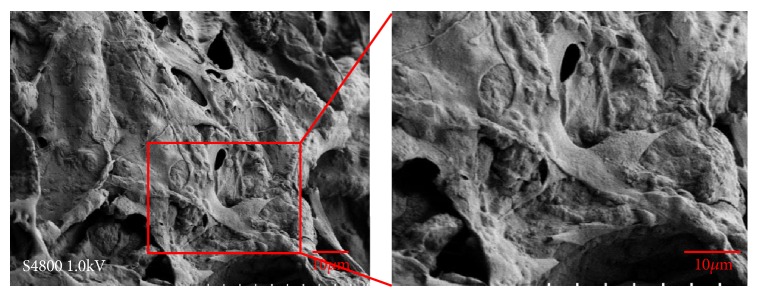
Scanning electron microscopy (SEM) of BMMSCs seeded onto nHACM/B9 scaffolds after 72 h of incubation. SEM images show a random distribution of cells on the surface of the nHACM/B9 scaffold.

To further research the cytotoxicity of scaffolds, CCK-8 assay was used. And the CCK-8 assay is a general method to evaluate the cytotoxicity of scaffolds in bone tissue engineering. In our study, OD values of the three groups indicated no statistically significant differences (Figure 3). Compared with BMMSCs cultured without scaffolds in cell culture plates, the cell activity of BMMSCs cultured on the two scaffolds was not decreased. BMMSCs grew on nHACM and nHACM/B9 scaffolds without cytotoxicity. In addition, SEM images showed cells randomly distributed on the surface or inside of the nHACM scaffolds. At 72 h, some cells had adhered to the surface and pores of the scaffold. SEM showed that single round cells became flat, polygonal, or triangular. Cell microfilaments and pseudopodia were tightly connected to the scaffold (Figure 4).

### 3.2. nHACM/B9 Scaffolds Promote Osteogenic Differentiation of BMMSCs In Vitro

**Figure 5:**
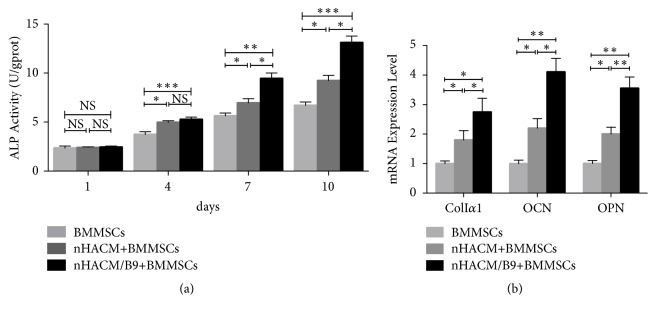
nHACM/B9 scaffolds promote osteogenic differentiation of BMMSCs* in vitro. *(a) There was a significant difference between the BMMSC group and nHACM and nHACM/B9 groups after 4 days of culture. (b) qPCR analysis of osteogenic marker genes indicated that BMMSCs cultured on nHACM scaffolds had increased expression of ColI*α*1, OCN, and OPN that are important genes to evaluate osteogenic differentiation of BMMSCs. Mean±SD; n=3; *∗p*<0.05, *∗∗p*<0.01, and *∗∗∗p*<0.001.

The experiments have testified that nHACM/B9 scaffold possessed good physicochemical properties and biocompatibility. The study to evaluate the ability of promoting osteogenic differentiation of BMMSCs* in vitro* was carried out. ALP activity is an important consideration for evaluating osteoblast differentiation. ALP activity was significantly increased in cells cultured on nHACM and nHACM/B9 compared with the control group (Figure 5(a)). qPCR showed that OCN, ColI*α*1, and OPN gene expression was increased in the nHACM/B9 group compared with the nHACM group (Figure 5(b)). And nHACM/B9 group was the highest of all. The results indicated that nHACM enhances osteoblast differentiation of BMMSCs* in vitro*, and the effect increases after BMP-9 loading.

### 3.3. nHACM/B9 Scaffolds Improve Bone Regeneration In Vivo

**Figure 6:**
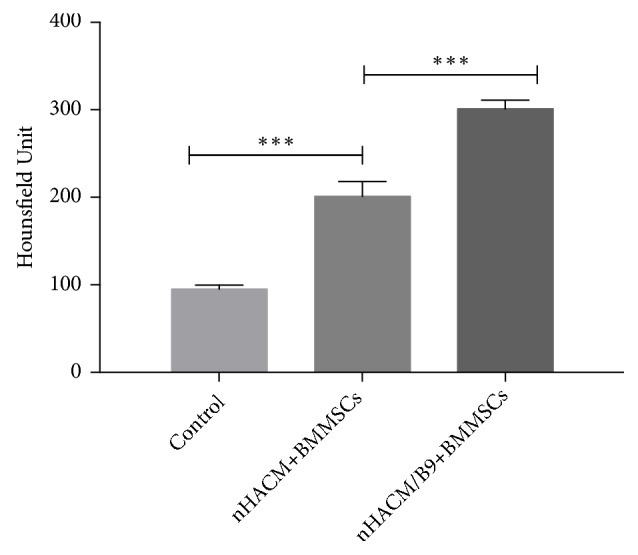
nHACM/B9 scaffolds reinforce bone regeneration* in vivo*. CT values of cranial bone specimens in the control, nHACM+BMMSC, and nHACM/B9+BMMSC groups. Mean±SD; n=3; *∗p*<0.05, *∗∗p*<0.01, and *∗∗∗p*<0.001.

**Figure 7:**
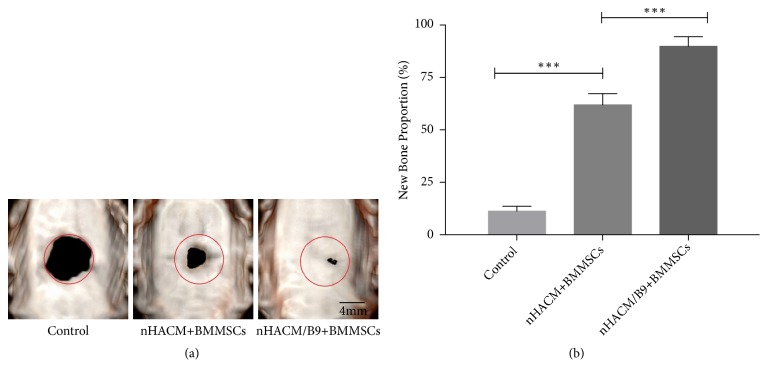
*In vivo* radiographic examinations at 12 weeks of repair. (a) Analysis of the CT images showed that the nHACM/B9+BMMSC group had a significantly larger area of new bone regeneration among the three groups. The nHACM+BMMSCs group had a significantly larger area of new bone regeneration than the control group. Red circles denote the original bony defects. (b) The proportion of newly formed bone in the control, nHACM+BMMSC, and nHACM/B9+BMMSC groups. Mean±SD; n=3; *∗p*<0.05, *∗∗p*<0.01, and *∗∗∗p*<0.001.

**Figure 8:**
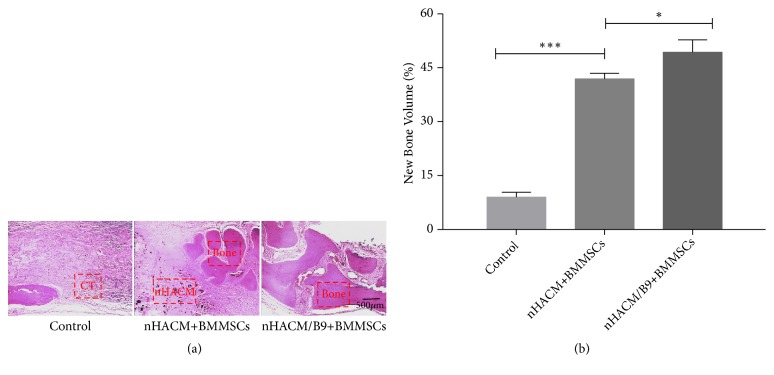
H&E staining on the sectioned slices at 12 weeks postoperatively. (a) There was new bone regeneration (Bone) in nHACM+BMMSC and nHACM/B9+BMMSC groups. Some fragments of nHACM were not completely biodegradable (nHACM). (b) New bone volumes of nHACM+BMMSC and nHACM/B9+BMMSC groups were much higher than the control group. There was connective tissue (CT) in the control group. Mean±SD; n=3; *∗p*<0.05, *∗∗p*<0.01, and *∗∗∗p*<0.001.

To further evaluate the efficacy of improving bone regeneration* in vivo,* an 8 mm CSD in SD rats was used. All 15 rats survived the surgery and recovered well postoperatively, without infection. Cranial bones samples were harvested at 12 weeks after implantation and analyzed by radiology and histology analysis. The HU of cranial bones samples showed that the nHAC/B9+BMMSC group had the significantly highest, and the HU of the nHACM+BMMSC group was higher than the control group (Figure 6). Analysis of CT images by software ImageJ showed that the nHACM/B9+BMMSC group had the significantly largest area of new bone regeneration, and the nHACM+BMMSCs group had a larger area of new bone regeneration than the control group (Figure 7) (Supplementary Figure D). Histological analysis confirmed that new bone tissue had formed in nHACM/B9+BMMSCs and nHACM+BMMSCs groups with a difference reflected in quantity (Figure 8). That indicated that the scaffold could stimulate new bone formation, and the effect will be enhanced after loading with BMP-9.

## 4. Discussion

In 1993, Langer and Vacanti [26] published an article entitled “Tissue engineering” in* Science*, which first proposed the basic implication of tissue engineering. As a development in bone tissue engineering, cells cultured with a specific scaffold to construct composite materials to repair bone defects have achieved initial success. It is an urgent task to develop suitable scaffolds with good physiochemical properties and biocompatibility for seeded cells in bone tissue engineering. An ideal scaffold should possess optimal mechanical properties, biocompatibility, and architecture for cell colonization and organization, ensuring the integration of the scaffold with bone tissue [27]. In several previous studies, nHA and ColI were blended together to effectively combine their beneficial properties. In recent years, carbon-based nanomaterials, such as CNTs, carbon nanohorns, and carbon nanodots, have attracted extensive attention as important candidates in the fields of nanomedicine and tissue engineering for their nano-size scale, their high electrical conductivity, and their unique mechanical strength [28, 29]. Cheng et al. [30] previously reported that the incorporation of CNT into PLGA scaffolds facilitates both the proliferation and osteogenic differentiation of MC3T3-E1 osteoblasts. In our study, nHA, ColI, and MWCNT were mixed to develop a new 3D scaffold that provided unique chemical, structural, and mechanical properties for bone tissue engineering and regeneration applications. MWCNT was used to enhance the mechanical strength and biocompatibility of the scaffold [31].

For tissue engineering scaffolds, adequate porosity, a suitable pore size, and interconnecting pores are important for in-growth, cellular adherence, and metabolite transport of cells [32, 33]. In present study, nHACM scaffolds had interconnected pores and a high degree of porosity. Pore sizes of nHACM scaffolds were between 88 and 153 *μ*m, which are an ideal biomaterial to interact and integrate with bone tissue. With the proportion of MWCNT increasing, the compressive strength and compressive modulus of nHACM scaffolds increased, but pore size, porosity, and water absorption decreased. Considering the above characterization, the most appropriate proportion of MWCNT, which is the most suitable for bone tissue engineering, was 1.0%.

There have been considerable investigations into the cytotoxicity of CNTs, with discrepancies reported in terms of its biological effects [34]. For instance, Bottini et al. [35] studied the toxicity of CNTs on human T cells and observed that CNTs could induce T lymphocyte apoptosis in a dose-dependent manner. However, Song et al. [36] found that HA-MWCNTs have a favorable biocompatibility, with no obviously detrimental effects on bone marrow-derived stem cells. The CCK-8 assay was used to evaluate the proliferation of cells seeded on the scaffolds and in the control group* in vitro*. The activity of cells was demonstrated through absorbance readings of the groups. The 0.5%MWCNT scaffolds exhibited similar proliferative activities to the 1%MWCNT and 1.5%MWCNT scaffolds, with no significant difference in the results. Therefore, taking into consideration the results of the physical characterization and CCK-8 assay, nHACM scaffolds containing 1.0% wt MWCNT were used for subsequent experiments. Compared with BMMSCs cultured without scaffolds in cell culture plates, the cell activity of BMMSCs cultured on the nHACM and nHACM/B9 scaffolds was not decreased. It indicated that nHACM and nHACM/B9 scaffolds were without cytotoxicity to BMMSCs. SEM images showed BMMSCs to be well spread out and integrated within the porous scaffolds, with fully protruded filopodia, confirming that the 3D scaffolds were conducive to cellular anchorage and guided cytoskeletal extensions. It is important to note that a similar phenomenon has been observed in other studies. Valverde et al. [37] showed that biocomposite scaffolds, fabricated by incorporating MTA and/or MWCNT into ColI scaffolds, promoted MC3T3-E1 adhesion, viability, and proliferation. BMP-9 was added to fabricate BMP-9-releasing nHACM scaffolds for bone tissue engineering. The BMP superfamily plays an important role in bone tissue engineering because of their effective osteogenic functions. BMPs are essential in various stages of bone healing [38]. Previous studies have focused on promoting osteogenesis by incorporating BMP-2, which has been shown to be strongly osteoinductive. However, recent studies have found BMP-9 to be more osteogenic than BMP-2 both* in vitro* and* in vivo* [39, 40]. Teven et al. [41] investigated the osteogenic potential of BMP-9 on mesenchymal progenitor cells using a recombinant BMP-9-expressing adenovirus (adBMP-9) system and found that BMP-9 effectively facilitates both osteogenic differentiation and new bone formation. Previous studies have employed adenovirus-mediated BMP gene therapy, but there are side effects such as tumorigenesis and immunogenicity [42]. Using biomaterials as carriers may provide controlled and sustained delivery of a growth factor and mimic the temporal profile during bone healing* in vivo* [43]. ColI and MWCNT, which are widely used as drug delivery systems in tissue engineering and pharmacology [44, 45], were employed together to develop a new system to release BMP-9 in this study.* In vitro*, analysis of ALP activity indicated that nHACM conferred a stimulatory effect on the differentiation of osteoblasts, especially when loaded with BMP-9. In addition, we found a significant elevation in the expression of osteogenesis-related markers in both nHACM and nHACM/B9 groups when compared with the control group, demonstrating that the composite scaffold can effectively induce the osteogenic differentiation of BMMSCs, even in the absence of BMP-9.

As reported in a previous study, the honeycomb-like scaffolds developed by the combination of poly (D, L-lactic acid) (PDLLA) and MWCNT could effectively promote osteogenic differentiation of osteoblast-like MG-63 cells [46]. Jing et al. [47] characterized composites of ColI-HA and MWCNT-ColI-HA and found that the inclusion of MWCNT increased new bone formation in calvarial bone defects. In the present study, BMMSCs were seeded on nHACM and nHACM/B9 scaffolds, and ColI and MWCNT were employed as the carrier to deliver BMP-9, which were implanted into critically sized rat calvarial bone defects. Blanks (no scaffold and no BMMSCs) were used as controls. CT results showed that the HU of the nHACM/B9+BMMSC group was much higher than that of the other groups. Analysis of CT images also showed that the effect of the nHACM/B9+BMMSCs group was better than that of other groups. These results indicated that the density and area of tissue regenerated in the nHACM/B9+BMMSCs group was the highest, and the osteogenic activity of the nHACM/B9+BMMSC group was the strongest with almost complete closure of bony defects. Histological analysis complemented the CT results. Compared with untreated control defects, nHACM and nHACM/B9 scaffolds with cells enhanced defect closure and mineralization compared with untreated control defects, indicating that the nHACM scaffold itself is osteoconductive. After loading with BMP-9, it can provide an even more effective approach to repair bone defects. Notably, BMP-9 promoted the effect of nHACM scaffolds that possess the potential for use in bone tissue engineering and correcting malunion of fractures. Interestingly, in some specimens, we found a small amount of MWCNT scattered around the new bone at the 12-week post-operative time point. This may be because MWCNT can functionalize as a specific nucleation site and effectively stimulate the initial crystallization of HA. In particular, Xiao et al. [48] demonstrated that MWCNTs acted as core for the growth and nucleation of the apatite crystallites.

## 5. Conclusions

In this study, a nHACM scaffold was developed by blending and freeze drying, and the proportion of 1.0% wt MWCNT was ideal for bone tissue engineering. The nHACM scaffold was biocompatible and showed no negative effects on rat BMMSCs* in vitro*. After loading with BMP-9, effects on promoting osteoblast differentiation* in vitro* and bone formation* in vivo* were stronger. This scaffold appears to be a suitable candidate for use in bone tissue engineering.

## Figures and Tables

**Table 1:** Primer sequences of osteogenic markers.

	forward primer	reverse primer
OCN	5′-TCTTTCTCCTTTGCCTGGC-3′	5′-CACCGTCCTCAAATTCTCCC-3′
ColI*α*1	5′-GCAACAGTCGCTTCACCTACA-3′	5′-CAATGTCCAAGGGAGCCACAT-3′
OPN	5′-TATCCCGATGCCACAGATGA-3′	5′-TGAAACTCGTGGCTCTGATG-3′
GAPDH	5′-TGTGTCCGTCGTGGATCTGA -3′	5′-TTGCTGTTGAAGTCGCAGGAG-3′

## Data Availability

The data used to support the findings of this study are included within the article.

## Supplementary Materials

Supplementary Materials Supplementary Figure: (A) To investigate the optimal content of BMP-9 loaded in each nHACM scaffold, ALP activity assay was conducted. BMMSCs were inoculated into the scaffolds with various concentrations of BMP-9 (250ng, 500ng and 750ng). After co-cultured for 1, 4, 7, and 10 days, there was no significant difference between 500ng group and 750ng group, while 500ng group was higher than 250ng group. Mean±SD; n=3; *∗p*<0.05, *∗∗p*<0.01, and *∗∗∗p*<0.001. (B) CCK-8 assays for** c**ell proliferation of BMMSCs that cultured on nHACM scaffolds with various concentrations (0.5, 1, and 1.5% wt). There were no significant differences in cell proliferation among the three groups. Mean±SD; n=3. (C) Cell morphology of BMMSCs in passage 3 examined under an inverted light microscope after cultured for 1, 2, 3, and 6 days. Most of the BMMSCs deformed as triangular or polygonal, and cells density was increasing obviously. (D) The blue parts represent the incomplete closure of the bone defects; the red parts represent the new bone regeneration. The new bone proportion means the red area minus the blue area.
