# Supplementary material for: Acceleration of Bone Regeneration in Critical-Size Defect Using BMP-9-Loaded nHA/ColI/MWCNTs Scaffolds Seeded with Bone Marrow Mesenchymal Stem Cells
Source: Biomed Res Int. 2019 Apr 11;2019:7343957. doi: 10.1155/2019/7343957 (PMC6487171; doi:10.1155/2019/7343957)
Supplement: Supplementary Materials — Supplementary Figure: (A) To investigate the optimal content of BMP-9 loaded in each nHACM scaffold, ALP activity assay was conducted. BMMSCs were inoculated into the scaffolds with various concentrations of BMP-9 (250ng, 500ng and 750ng). After co-cultured for 1, 4, 7, and 10 days, there was no significant difference between 500ng group and 750ng group, while 500ng group was higher than 250ng group. Mean±SD; n=3; ∗p<0.05, ∗∗p<0.01, and ∗∗∗p<0.001. (B) CCK-8 assays for cell proliferation of BMMSCs that cultured on nHACM scaffolds with various concentrations (0.5, 1, and 1.5% wt). There were no significant differences in cell proliferation among the three groups. Mean±SD; n=3. (C) Cell morphology of BMMSCs in passage 3 examined under an inverted light microscope after cultured for 1, 2, 3, and 6 days. Most of the BMMSCs deformed as triangular or polygonal, and cells density was increasing obviously. (D) The blue parts represent the incomplete closure of the bone defects; the red parts represent the new bone regeneration. The new bone proportion means the red area minus the blue area. [file 7343957.f1.pdf]

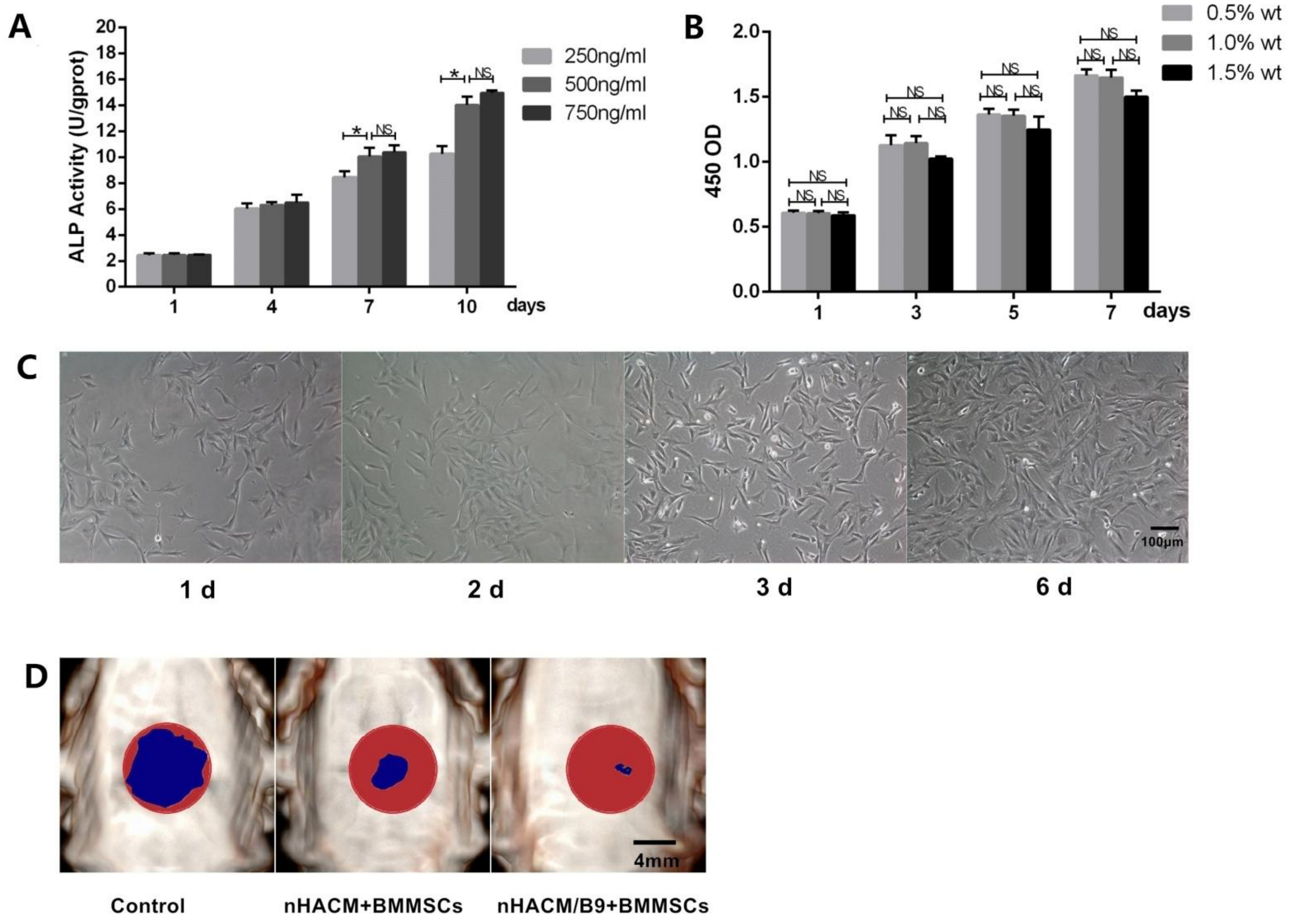

Supplementary Figure

Supplementary Figure: (A) To investigate the optimal content of BMP-9 loaded in each nHACM scaffold, ALP activity assay was conducted. BMMSCs were inoculated into the scaffolds with various concentrations of BMP-9 (250ng, 500ng and 750ng). After co-cultured for 1, 4, 7, and 10 days, there was no significant difference between 500ng group and 750ng group, while 500ng group was higher than 250ng group. Mean±SD; n=3; **p*<0.05, ***p*<0.01, and ****p*<0.001. (B) CCK-8 assays for **c**ell proliferation of BMMSCs that cultured on nHACM scaffolds with various concentrations (0.5, 1, and 1.5% wt). There were no significant differences in cell proliferation among the three groups. Mean±SD; n=3. (C) Cell morphology of BMMSCs in passage 3 examined under an inverted light microscope after cultured for 1, 2, 3, and 6 days. Most of the BMMSCs deformed as triangular or polygonal, and cells density was increasing obviously. (D) The blue parts represent the incomplete closure of the bone defects, the red parts represent the new bone regeneration. The new bone proportion means the red area minus the blue area.
